# Prophylactic drainage versus non-drainage following gastric cancer surgery: a meta-analysis of randomized controlled trials and observational studies

**DOI:** 10.1186/s12957-023-03054-1

**Published:** 2023-06-03

**Authors:** Hua-Yang Pang, Li-Hui Chen, Xiu-Feng Chen, Meng-Hua Yan, Zhi-Xiong Chen, Hao Sun

**Affiliations:** 1 Gastrointestinal Cancer Center, Chongqing University Cancer Hospital, Chongqing, China; 2 Chongqing Key Laboratory of Translational Research for Cancer Metastasis and Individualized Treatment, Chongqing University Cancer Hospital, Chongqing, China

**Keywords:** Gastric cancer, Prophylactic drainage, Non-drainage, Gastrectomy, Meta-analysis

## Abstract

**Background:**

The role of prophylactic drainage (PD) in gastrectomy for gastric cancer (GC) is not well-established. The purpose of this study is to compare the perioperative outcomes between the PD and non-drainage (ND) in GC patients undergoing gastrectomy.

**Methods:**

A systematic review of electronic databases including PubMed, Embase, Web of Science, the Cochrane Library, and China National Knowledge Infrastructure was performed up to December 2022. All eligible randomized controlled trials (RCTs) and observational studies were included and meta-analyzed separately. The registration number of this protocol is PROSPERO CRD42022371102.

**Results:**

Overall, 7 RCTs (783 patients) and 14 observational studies (4359 patients) were ultimately included. Data from RCTs indicated that patients in the ND group had a lower total complications rate (OR = 0.68; 95%CI:0.47–0.98; *P* = 0.04; *I*^2^ = 0%), earlier time to soft diet (MD =  − 0.27; 95%CI: − 0.55 to 0.00; *P* = 0.05; *I*^2^ = 0%) and shorter length of hospital stay (MD =  − 0.98; 95%CI: − 1.71 to − 0.26; *P* = 0.007; *I*^2^ = 40%). While other outcomes including anastomotic leakage, duodenal stump leakage, pancreatic leakage, intra-abdominal abscess, surgical-site infection, pulmonary infection, need for additional drainage, reoperation rate, readmission rate, and mortality were not significantly different between the two groups. Meta-analyses on observational studies showed good agreement with the pooled results from RCTs, with higher statistical power.

**Conclusion:**

The present meta-analysis suggests that routine use of PD may not be necessary and even harmful in GC patients following gastrectomy. However, well-designed RCTs with risk-stratified randomization are still needed to validate the results of our study.

**Supplementary Information:**

The online version contains supplementary material available at 10.1186/s12957-023-03054-1.

## Background

Gastric cancer (GC) is one of the most common causes of cancer-related deaths worldwide [1, 2]. Despite encouraging advances in chemoradiotherapy, targeted therapy, and immunotherapy, surgery remains the cornerstone of treatment for GC. Gastrectomy is regarded as a technically demanding abdominal surgery, with considerable postoperative complications rate, such as anastomotic leakage, bleeding, and intra-abdominal abscess [3, 4].

Prophylactic drainage (PD) has long been routinely performed in abdominal surgery for the purpose of preventing and managing potential postoperative abdominal complications [5, 6]. However, as related research advances, evidence is accumulating that PD may not be as clinically valuable as thought [7, 8]. A previous study involving 17 randomized controlled trials (RCTs) demonstrated that PD did not contribute to the reduction of morbidities following colorectal surgery, appendectomy, hepatectomy, and cholecystectomy [9]. In this context, avoidance of PD is strongly recommended for inclusion in the enhanced recovery after surgery (ERAS) pathway for GC surgery.

However, evidence for avoiding routine PD after surgery in patients with gastric cancer is sparse. In 2020, a meta-analysis based on 10 studies concluded that PD avoidance may favor a reduction in morbidities and a trend towards a decrease in length of stay [10]. Nevertheless, these pooled results were based on only 3 RCTs, which were not in line with those derived from observational studies. Also, the role of PD in other important perioperative outcomes was not well elucidated due to limited data. As a series of new RCTs and observational studies have been published over the years, we aim to perform an updated meta-analysis based on existing evidence to investigate the role of PD in GC patients after gastrectomy.

## Methods

Our meta-analysis was performed in line with the requirements from PRISMA (Preferred Reporting Items for Systematic Reviews and Meta-Analyses) guidelines [11] and assessing the methodological quality of systematic reviews (AMSTAR) Guidelines [12]. The meta-analysis was registered in PROSPERO (CRD42022371102).

### Search strategy

Relevant studies from electronic datasets including PubMed, Embase, Web of Science, the Cochrane Central Register of Controlled Trials, and China National Knowledge Infrastructure were systematically examined up to December 15, 2022. The following key words (limited to title or abstract) were combined with Boolean operators AND or OR, to comprehensively capture potential articles: “drainage,” “drain,” “gastric cancer,” “gastric carcinoma,” “stomach cancer,” and “stomach neoplasm”. The search strategy was applied to suit each database, and the complete search strategy was reported in the supplementary file: Table S1. During the search process, language restrictions were not applied. In addition, the references of the included studies were manually searched for additional reports. The search was performed by two investigators independently (HY-P and LH-C).

### Inclusion and exclusion criteria

The inclusion criteria were determined according to the PICOS approach as follows. *P*: Patients were pathologically diagnosed with GC and underwent gastrectomy; *I*: non-drainage; *C*: prophylactic drainage; *O*: perioperative outcomes; *S*: Comparative studies including RCTs, cohort and case-controlled studies.

The exclusion criteria were studies (1) reported as case reports, reviews, letters, and abstracts and (2) with overlapping data.

### Data extraction

Two independent reviewers (HY-P and LH-C) conducted the data extraction and cross-checked all the results, and any discrepancies were resolved by a third reviewer (H S). The following data were extracted from each study: first author, publication year, study interval, country, study design and sample size, age, sex, neoadjuvant therapy, surgical approach, gastrectomy extent, combined organ resection, surgical margin, lymphadenectomy extent, TNM stage, time of drainage removal, and a series of perioperative outcomes.

### Quality assessment and certainty of evidence assessment

The Cochrane Risk-of-Bias 2.0 (RoB 2.0) [13] tool was used to assess the risk of bias for RCTs, from five domains: randomization process, deviations from intended interventions, missing outcome data, measurement of the outcome, and selection of the reported result. While the Risk of Bias in Non-Randomized Studies-of Interventions (ROBINS-I) [14] tool was used to assess the risk of bias for observational studies, from seven domains: confounding factors, selection of participants into the study, classification of interventions, deviations from intended interventions, missing data, measurement of outcomes, and selection of the reported results. Regarding the quality of evidence of each outcome, the Grading of Recommendations Assessment, Development, and Evaluation (GRADE) [15] approach was applied, which scores each endpoint from very low to high.

### Outcomes of interest and definitions

Perioperative outcomes that occur during hospitalization or within 30 days after surgery were assessed in this study, including total complications, anastomotic leakage, duodenal stump leakage, pancreatic leakage, intra-abdominal abscess, surgical-site infection, pulmonary infection, need for additional drainage, time to first soft diet, length of hospital stay, reoperation, readmission, mortality and drain-related complications.

### Statistical analysis

The odds ratios (ORs) and mean differences (MDs) with their 95% confidence intervals (CIs) were used as the effect sizes for dichotomous variables and continuous variables, respectively. For studies that reported median with range or inter-quartile range, data were converted into mean with standard deviation (SD) using the method reported by McGrath et al. [16]. Heterogeneity among studies was assessed using I^2^ statistic. In the present study, all meta-analyses were performed assuming the random-effects model, which accounts for variance across included studies. Subgroup group analysis and meta-regression analysis were performed to investigate the sources of heterogeneity. Publication bias was tested using Begg’s funnel plot when there were at least 10 studies included. A two-tailed *P* value < 0.05 was considered statistically significant. All of these statistical analyses were performed by Review Manager Software, version 5.3 (Cochrane, London, UK), and Stata, version 12.0 (Statacorp, College Station, TX).

## Results

### Study characteristics

**Fig. 1:**
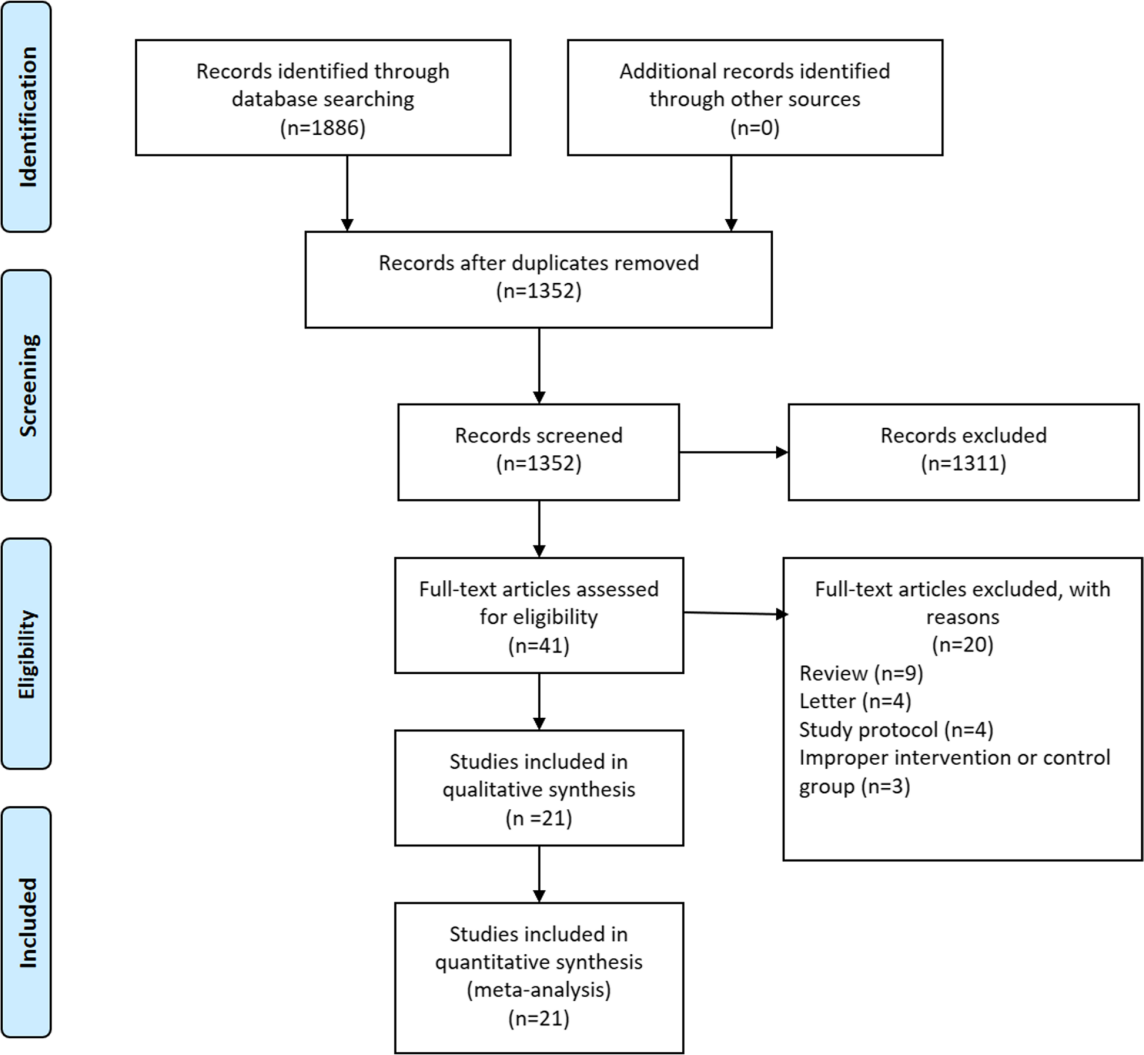
The PRISMA Flowchart of study selection

A flow chart of the selection process was shown in Fig. 1. The search strategy yielded 1886 potential studies. After the title, abstract, and full text assessment, 7 RCTs [17–23] and 14 observational [24–37] studies were finally included in the present study. The basic features of the 21 studies involved were shown in Table 1. A total of 5142 GC patients were included in this study. These studies were from 8 countries and published between 2004 and 2022, with a sample size ranging from 21 to 1989. Among these studies, 5 of them included patients who underwent neoadjuvant therapy. Open and minimally invasive gastrectomy were both performed in these patients. Based on the extent of tumor involvement, these patients underwent either distal, proximal, subtotal, or total gastrectomy regardless of curative resection or not. Combined organ resection was performed when necessary. The criteria of removing the drainage tubes also varied a lot among included studies. Additionally, the incidence of drain-related complications of included studies ranged from 1.5% to 9.4%, mainly including drain site infection, continuous leakage, and omentum coming out.

**Table 1:** Basic information of included studies

References	Country	Study design	Sample size (no drain: drain)	Study interval	Sex (M/F)	Age, year	Neoadjuvant therapy (%)	Surgical approach	Gastrectomy extent	Combined organ resection	Surgical margin	Lymphadene ctomy	TNM stage	Drainage removal	Drain-related complications (%)
Kim, 2004 [20]	Korea	RCT	170 (84:86)	2001–2001	116/52	NA	NA	NA	STG; TG	Yes	R0	D2/D2 +	I–IV	Drainage was less than 100 ml	NA
Alvarez Uslar, 2005 [17]	Chile	RCT	60 (32:29)	2000–2003	43/17	61 (range 36–79)	NA	NA	TG	Yes	Any R	D2	NA	Usually 8 days after surgery	NA
Kumar, 2007 [30]	Nepal	Observational	108 (52:56)	2001–2005	69/39	55.6 ± 15.7	NA	NA	STG	No	Any R	D1/D2	I–IV	Drainage was clear and less than 50 ml	7.1
Jiang, 2008 [19]	China	RCT	100 (51:49)	2005–2006	59/41	NA	0	NA	DG; PG; TG	No	R0	D2	I–III	Drainage was clear and less than 100 ml	2.0
Li, 2008 [32]	China	Observational	67 (35:32)	2003–2007	53/14	62.0 ± 11.0	NA	NA	DG	NA	R0	D1/D2	NA	NA	9.4
Zhang, 2009 [37]	China	Observational	311 (66:245)	2007–2008	233/78	58.9 ± 11.8	NA	NA	DG; PG; TG	NA	R0	D1/D2	I–IV	8.3 ± 2.2 day	NA
Zhang, 2010 [23]	China	RCT	67 (35:32)	NA	53/14	61.6 ± 11.1	NA	NA	DG	NA	R0	D1/D2	NA	NA	9.4
Chen, 2011 [18]	China	RCT	130 (65:65)	2009–2011	43/22	62.1 ± 3.2	NA	NA	NA	NA	R0	NA	NA	NA	NA
Fu, 2011 [27]	China	Observational	117 (13:104)	2009–2010	86/31	57.8 ± 10.9	0	NA	DG; PG	NA	R0	D1/D2	I–III	NA	NA
Ishikawa, 2011 [29]	Japan	Observational	21 (11:10)	2004–2008	15/6	NA	NA	Laparoscopic	DG	No	NA	D1 + a	NA	Usually 4 or 5 days after surgery	NA
Song, 2011 [22]	China	RCT	148 (74:74)	2006–2009	117/31	< 75	0	NA	DG; PG; TG	NA	R0	D2	I–IV	NA	NA
Cai, 2013 [25]	China	Observational	250 (40:210)	2005–2011	155/95	58.9 ± 11.8	NA	NA	NA	NA	NA	NA	NA	NA	NA
Dann, 2015 [26]	USA	Observational	344 (91:253)	2000–2012	200/144	65 (range 24–92)	30.0	Open/laparoscopic	TG	Yes	R0/1	D0–D3	I–III	NA	NA
Hirahara, 2015 [28]	Japan	Observational	78 (33:45)	2011–2014	51/27	NA	NA	Laparoscopic	DG	NA	R0	D1 + /D2	I–III	NA	NA
Lee, 2015 [31]	Korea	Observational	1989 (740:1249)	2012–2013	1266/723	NA	0	Open/laparoscopic/robotic	STG; TG	Yes	R0	D1 + /D2	I–III	NA	NA
Schots, 2018 [35]	Netherlands	Observational	107 (40:67)	2013–2017	62/45	NA	66.4	Open/laparoscopic	DG; TG	No	R0	D2	NA	Drainage was clear and less than 150 ml	1.5
Akira, 2019 [24]	Japan	Observational	94 (65:29)	2007–2014	62/32	70	NA	Laparoscopic	TG	NA	NA	D1/D1 + /D2	I–III	Drainage was less than 100 ml	NA
Shimoike, 2019 [36]	Japan	Observational	290 (145:145)	2011–2017	199/91	NA	6.2	Laparoscopic	DG; TG	Yes	any R	D1 + or less/D2	NA	NA	NA
Lim,2020 [33]	Korea	Observational	499 (111:388)	2010–2017	353/146	62.2 ± 12.0	NA	Open/laparoscopic	TG	Yes	NA	D1 + /D2	I–IV	Usually 4 or 5 days after surgery	NA
Liu, 2021 [34]	China	Observational	84 (42:42)	2018–2019	60/24	NA	7.0	Laparoscopic	DG	NA	NA	NA	I–III	NA	NA
Muduly, 2022 [21]	India	RCT	108 (54:54)	NA	77/31	56.4 ± 11.3	32.4	NA	DG; PG; STG; TG	NA	R0	D2	I–IV	Drainage was clear and less than 200 ml	NA

*M* Male, *F* Female, *RCT* Randomized controlled trial, *MIS* Minimally invasive surgery, *TG* Total gastrectomy, *STG* Subtotal gastrectomy, *PG* Proximal gastrectomy, *DG* Distal gastrectomy, *NA* Not available

### Meta-analysis of outcomes

#### Adverse event outcomes

**Fig. 2:**
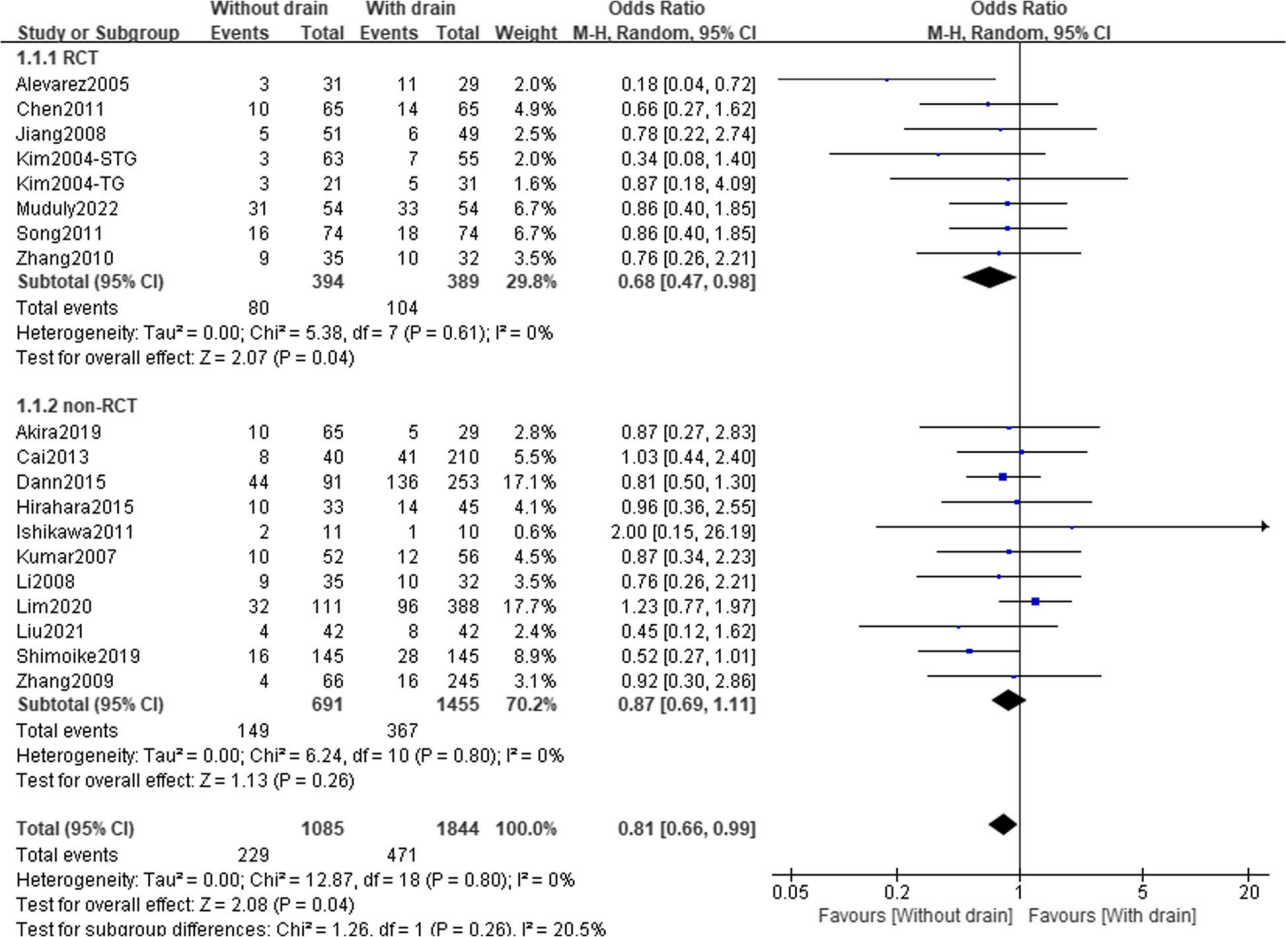
Forest plot assessing total complications rate between the PD and ND groups

All seven RCTs including 783 patients and 11 observational studies including 2146 patients contributed data for total complications (Table 2 and Fig. 2). The pooled analysis deriving from RCTs demonstrated that patients in the ND group had a 32% lower risk of total complications than the PD group (OR = 0.68; 95%CI:0.47–0.98; *P* = 0.04; *I*^2^ = 0%); observational data showed concordant result, however, without significant difference (OR = 0.87; 95%CI:0.69–1.11; *P* = 0.26; *I*^2^ = 0%). In addition, we compared the incidence of specific complications between the ND and PD groups. As shown in Table 2 and Fig. S1, the distribution of all reported specific complications was not significantly different between the two groups, either in the RCT subset or the observational subset.

**Table 2:** Perioperative outcomes of gastric cancer patients between the PD and ND groups

Variables	Study design	Studies, n	Patients, n	OR or MD (95%CI)	P value	*I*^2^ (%)	Publication bias (Begg’s *P* value)	Quality of evidence (GRADE)
**Total complications**	RCT	7	783	0.68 (0.47–0.98)	0.04	0		
	Observational	11	2146	0.87 (0.69–1.11)	0.26	0		
	Total	18	2929	0.81 (0.66–0.99)	0.04	0	0.600	Low ^a^
**Anastomotic leakage**	RCT	4	383	0.62 (0.15–2.49)	0.50	0		
	Observational	11	4010	0.95 (0.56–1.62)	0.76	0		
	Total	15	4393	0.90 (0.55–1.48)	0.69	0	0.755	Low ^a^
**Duodenal stump leakage**	RCT	2	168	0.33 (0.05–2.22)	0.25	0		
	Observational	5	674	1.06 (0.21–5.34)	0.94	9		
	Total	7	842	0.67 (0.20–2.22)	0.51	0	-	Very low ^a,^ ^e^
**Pancreatic leakage**	RCT	1	60	0.30 (0.01–7.70)	0.47	-		
	Observational	5	1301	0.61 (0.19–1.93)	0.40	18		
	Total	6	1361	0.56 (0.21–1.49)	0.25	0	-	Very low ^a,^ ^e^
**Intra-abdominal abscess**	RCT	4	445	0.72 (0.25–2.04)	0.53	0		
	Observational	7	1219	1.26 (0.65–2.43)	0.50	0		
	Total	11	1664	1.07 (0.61–2.43)	0.81	0	0.451	Low ^a^
**Surgical-site infection**	RCT	5	593	0.77 (0.38–1.54)	0.45	0		
	Observational	7	1292	0.72 (0.40–1.32)	0.29	0		
	Total	12	1885	0.74 (0.47–1.17)	0.20	0	0.542	Low ^a^
**Pulmonary infection**	RCT	5	545	0.95 (0.39–2.28)	0.90	0		
	Observational	7	1055	0.80 (0.44–1.45)	0.46	0		
	Total	12	1600	0.84 (0.51–1.38)	0.50	0	0.502	Low ^a^
**Need for additional drainage**	RCT	2	278	1.07 (0.15–7.38)	0.95	0		
	Observational	3	2411	1.67 (0.38–7.23)	0.49	85		
	Total	5	2689	1.49 (0.45–4.90)	0.51	73	-	Very low ^a,^ ^b,^ ^e^
**Time to first soft diet**	RCT	4	438	− 0.27 (− 0.55–0.00)	0.05	0		
	Observational	6	698	− 0.78 (− 1.32 to − 0.24)	0.005	74		
	Total	10	1136	− 0.54 (− 0.85 to − 0.23)	0.0007	56	0.119	Very low ^a,^ ^b^
**Length of hospital stay**	RCT	6	716	− 0.98 (− 1.71 to − 0.26)	0.007	40		
	Observational	12	2303	− 0.43 (− 1.08–0.21)	0.19	61		
	Total	18	3019	− 0.63 (− 1.14 to − 0.12)	0.02	60	0.529	Very low ^a,^ ^b^
**Reoperation**	RCT	2	168	0.64 (0.23–1.84)	0.41	25		
	Observational	5	1146	0.57 (0.29–1.14)	0.11	0		
	Total	7	1314	0.61 (0.35–1.04)	0.07	0	-	Very low ^a,^ ^e^
**Readmission**	RCT	0	0	-	-	-		
	Observational	3	950	1.47 (0.87–2.47)	0.15	0		
	Total	3	950	1.47 (0.87–2.47)	0.15	0	-	Very low ^a,^ ^e^
**Mortality**	RCT	4	416	1.30 (0.14–11.92)	0.82	25		
	Observational	9	1881	0.56 (0.24–1.29)	0.17	0		
	Total	13	2297	0.65 (0.30–1.40)	0.27	0	-	Low ^a^

Downgrade quality of evidence: a: risk of bias; b: inconsistency; c: indirectness; d: imprecision; e: publication bias. Upgrade quality of evidence: f: large effect; g: plausible confounding would change the effect; h: dose–response gradient. *OR* Odds ratio, *MD* Mean difference, *CI* Confidence interval

Data on mortality were reported in 13 studies (4 RCTs involving 416 patients and 9 observational studies comprising 1881 patients). The pooled result from RCTs suggested that there was no evidence that ND increased the risk of early death (OR = 1.30; 95%CI:0.14–11.92; *P* = 0.82; *I*^2^ = 25%; Table 2 and Fig. S1). Besides, pooled data from observational studies indicated a trend towards lower mortality in the ND group (OR = 0.56; 95%CI:0.17–1.29; *P* = 0.17; *I*^2^ = 0%; Table 2 and Fig. S1).

#### Postoperative recovery outcomes

**Fig. 3:**
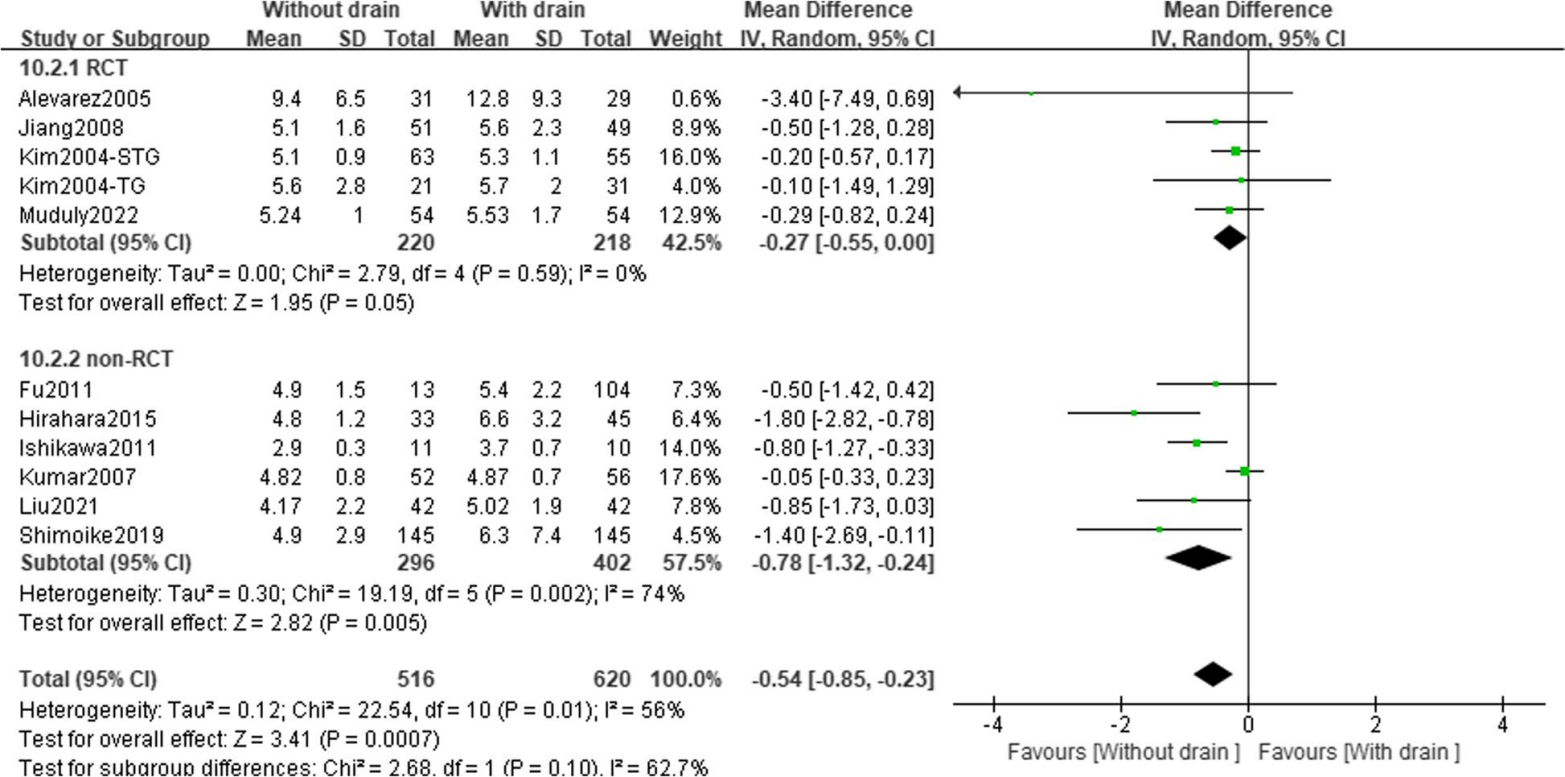
Forest plot assessing the time to first soft diet between the PD and ND groups

A total of 4 RCTs involving 438 patients and 6 observational studies involving 698 patients reported on time to first soft diet (Table 2 and Fig. 3). Pooled data from RCTs showed an earlier time to soft diet in the ND group (MD =  − 0.27; 95%CI: − 0.55 to 0.00; *P* = 0.05; *I*^2^ = 0%), which was in line with the results of observational studies (MD =  − 0.78; 95%CI: − 1.32 to − 0.24; *P* = 0.005; *I*^2^ = 74%). While the results of the heterogeneity test demonstrated a high heterogeneity among the observational studies.

**Fig. 4:**
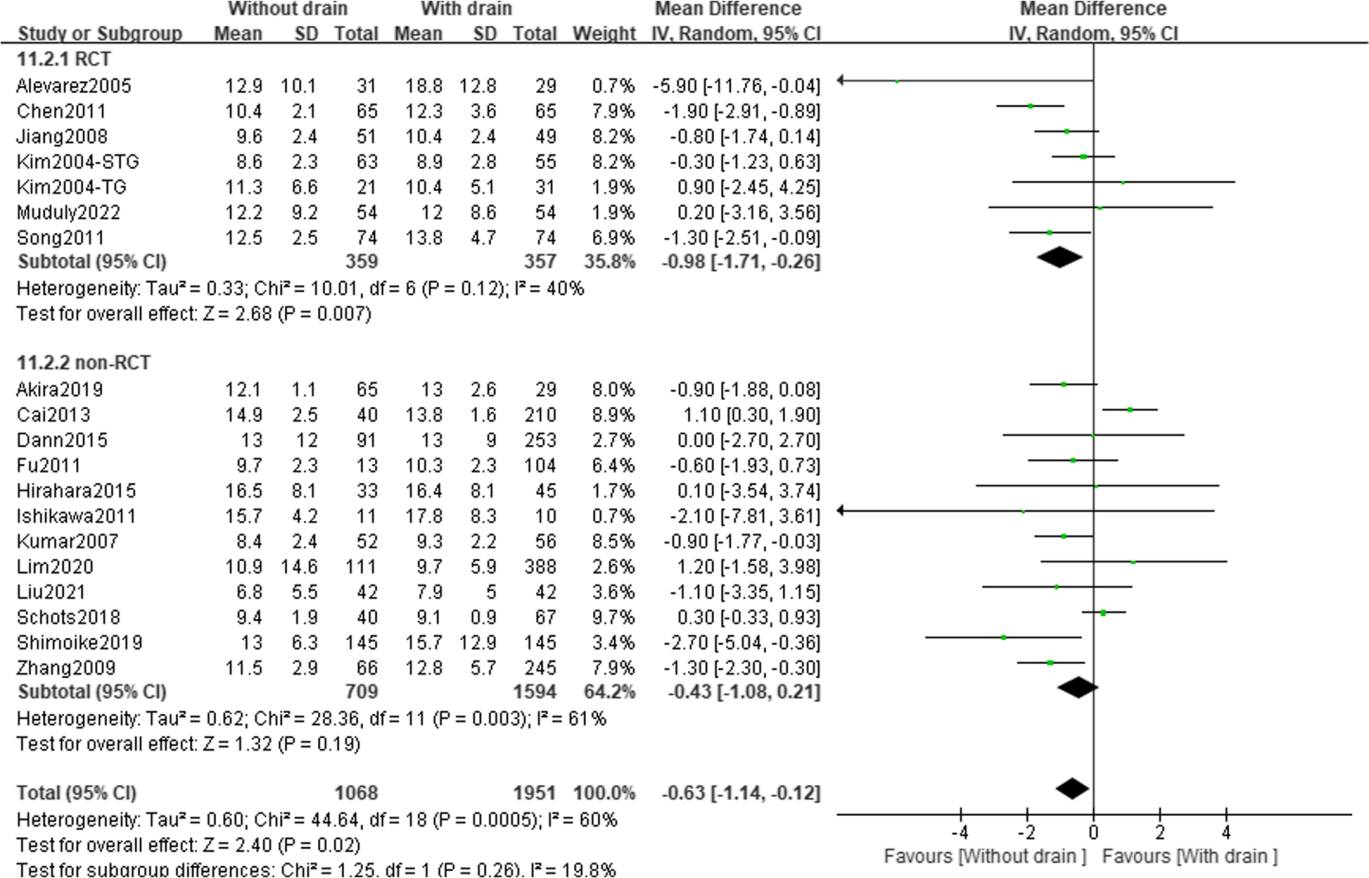
Forest plot assessing the length of hospital stay between the PD and ND groups

Data regarding postoperative hospital stay were available from 6 RCTs and 12 observational studies, including 716 and 2303 patients, respectively (Table 2 and Fig. 4). In the RCT subset, patients in the ND group showed a 0.98-day lower length of hospital stay than patients in the PD group (95%CI: − 1.71 to − 0.26; *P* = 0.007; *I*^2^ = 40%). The observational data were consistent, although the statistical difference threshold was not reached (MD =  − 0.43; 95%CI: − 1.08 to 0.21; *P* = 0.19; *I*^2^ = 61%). The results of the heterogeneity test demonstrated a moderate and high heterogeneity among the RCTs and observational studies, respectively.

#### Need for additional drainage, reoperation, and readmission

As shown in Table 2 and Fig. S1, there were 2 RCTs involving 278 patients and 3 observational studies including 2411 patients reporting the need for additional drainage. Pooled results from both the RCTs (OR = 1.07; 95%CI:0.15–7.38; *P* = 0.95; *I*^2^ = 0%) and observational studies (OR = 1.67; 95%CI:0.38–7.23; *P* = 0.49; *I*^2^ = 85%) demonstrated a similar rate of additional drainage between the ND and PD patients. However, the results of the heterogeneity test showed a high heterogeneity among the observational studies.

Two RCTs comprising 168 patients reported on reoperation. Pooled data from RCTs showed no significant difference in reoperation rate between the ND and PD groups (OR = 0.64; 95%CI:0.23–1.84; *P* = 0.41; *I*^2^ = 25%; Table 2 and Fig. S1). Across 5 observational studies comprising 1146 patients, the reoperation rate tended to be lower in the ND group (OR = 0.57; 95%CI:0.29–1.14; *P* = 0.11; *I*^2^ = 0%; Table 2 and Fig. S1).

No RCT was found eligible for evaluating the readmission rate in this study. Three observational studies, with 950 patients involved, demonstrated that there was no significant difference in terms of readmission rate between the two groups (OR = 1.47; 95%CI:0.87–2.47; *P* = 0.15; *I*^2^ = 0%; Table 2 and Fig. S1).

#### Subgroup analysis and meta-regression analysis

Subgroup analyses stratified by the sample size (≥ 100 vs. < 100) and academic institution (Yes vs. No) were performed to explore the potential discrepant treatment effect of different subgroups. Moreover, the efficacy of PD was also explored in GC patients who underwent laparoscopic surgery or a total gastrectomy. As shown in Fig. S2–5, the findings of all subgroup analyses demonstrated that the perioperative outcomes of the ND group were not inferior to the PD group. In addition, among patients undergoing laparoscopic gastrectomy, the incidence of anastomotic leakage and pancreatic leakage was slightly lower in the ND group. And in patients undergoing total gastrectomy, the reoperation rate (*P* = 0.06) tended to be lower in the ND group than in the PD group.

For pooled outcomes with significant heterogeneity (time to first soft diet and postoperative hospital stay), meta-regression analyses based on the following covariates were also performed to investigate the sources of heterogeneity: study design (RCT vs. non-RCT), sample size (≥ 100 vs. < 100), academic institution (Yes vs. No), surgical approach (laparoscopic gastrectomy or not) and surgical procedure (total gastrectomy or not). As shown in Table S2, for these pooled results, none of these variables contributed to the source of heterogeneity (all *P* values > 0.05).

### Risk of bias and certainty of evidence assessment

**Fig. 5:**
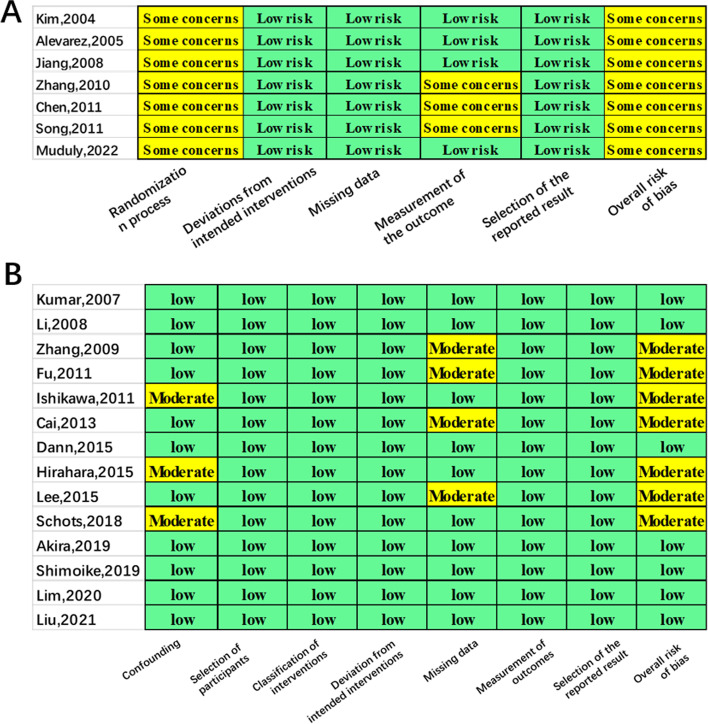
Risk of bias assessment for randomized control trials using the RoB 2.0 tool (**A**) and observational studies using the ROBINS-I tool (**B**)

As shown in Fig. 5A, all 7 RCT studies were evaluated using the RoB 2.0 tool and were of some concerns in the overall risk of bias. To be specific, three RCTs had some concerns in the domain of measurement of outcome, and all of them had some concerns in the domain of the randomization process because none of the studies reported specific implementation methods of randomization and allocation concealment. The 14 observational studies were evaluated using the ROBINS-I tool, and 7 of them were moderate risk in the overall risk of bias due to 3 studies had a moderate risk in the domain of confounding factors and 4 studies had a moderate risk in the domain of missing data (Fig. 5B). According to the GRADE approach, the overall certainty of the of evidence of each outcome was low or very low (Table 2).

### Publication bias

The Begg’s funnel plot was used to assess the potential publication bias of the pooled outcomes including at least 10 studies. As shown in Table 2 and Fig. S6, all of the *P* values were greater than 0.05, indicating that these pooled outcomes had a low risk of publication bias.

## Discussion

Currently, the routine placement of abdominal drainage tubes after gastrectomy is still widely used worldwide for the early diagnosis and management of critical abdominal complications such as post-operative bleeding, anastomotic leakage, and intra-abdominal infections [5]. Successive studies, however, have shown no clear benefit from prophylactic abdominal drainage [10, 21]. In addition, the placement of drainage tubes increases the patient’s postoperative pain, prolongs the use of analgesics and leads to the occurrence of drainage-related complications [20]. As a result, some institutions no longer routinely perform PD after GC surgery. Nevertheless, as these studies are limited by relatively small sample sizes and underpowered statistics, the conclusions are unclear.

To our knowledge, this is the largest meta-analysis (21 studies including 5142 patients) to evaluate the role of PD in perioperative outcomes of GC surgery. In this study, we found that the routine use of PD after surgery did not reduce the incidence of abdominal complications such as anastomotic leakage and pancreatic leakage. In contrast, the overall complication rate was significantly higher in the PD group. In addition, the length of hospital stay and the time to soft diet were much longer in the PD group than in the ND group. Moreover, PD did not also show any benefit in reducing readmission, reoperation, or mortality in GC surgery.

Several previously published meta-analyses [10, 38–40] have demonstrated the potential benefits of PD avoidance in GC patients, which were largely in line with our results. However, those studies were only able to achieve reliable conclusions in a few variables due to a limited number of included studies. At variance, by integrating all applicable RCTs and observational studies, the present study highlighted a faster recovery in the ND group, except for a reduced morbidity and hospital stay, while the previous studies did not find this difference between the two groups. Moreover, benefiting from the increased sample size, nearly all the results in our study showed low heterogeneity and good agreement across the RCT subset and observational subset, further convincing us of the efficacy of ND in GC surgery.

In recent years, laparoscopic surgery has been widely performed in GC, but the role of PD in laparoscopic gastrectomy is still unclear. Therefore, we performed a subgroup analysis for laparoscopic resections. Based on the results from 567 patients who underwent laparoscopic gastrectomy, our finding of the benefit of ND in these patients remained unchanged. Besides, we found that in this subgroup, the incidence of anastomotic leakage (*P* = 0.11) and pancreatic leakage (*P* = 0.07) was slightly lower in the ND group, although there was no strong evidence at the pooled analyses that routine ND has an effect on reducing these adverse outcomes. With advances in surgical techniques and laparoscopic equipment, laparoscopic surgery has been shown to be less likely to result in serious postoperative complications in experienced centers, due to its minimally invasive nature [41–43]. Consequently, we believe that routinely using PD following laparoscopic gastrectomy is not necessary.

The avoidance of drainage tubes in simple and routine surgery is well understood, but its feasibility in the context of complex surgery is uncertain. Total gastrectomy is a highly complex and challenging surgical procedure in GC patients. Its operation time, intraoperative blood loss, and postoperative complications are much higher than other surgical methods [44, 45]. However, in our present analysis based on 1049 patients, we found that PD did not show any advantage over ND in patients undergoing total gastrectomy. Unexpectedly, several recent meta-analyses demonstrated that even pancreaticoduodenectomy and major liver resection can safely avoid abdominal drainage, which indicated that PD is not a substitute for a meticulous surgical procedure in complex operations [8, 46]. In view of this, avoiding routine drainage should also be recommended during total gastrectomy.

To further clarify the reliability and generalizability of our study, we also analyzed the effect of sample size (≥ 100 vs. < 100) and hospital nature (academic institution vs. non-academic institution) on the perioperative outcomes of PD in GC patients. As shown in Fig. S2–3, the pooled results of these subgroup analyses remained consistent with our previous meta-analyses. These results further convinced us that routine drainage after gastrectomy was not indispensable, even in non-academic hospitals where the surgeons’ expertise and the back system are relatively insufficient compared to academic hospitals.

Nevertheless, our findings are based on literature, some uncertainties exist in the evidence included in this meta-analysis. The lack of stratified information in the original literature prevented us from analyzing the applicability of ND in certain specific subgroups, such as patient demographics (age, BMI, co-morbidity, and history of abdominal surgery), surgical parameters (combined organ resection, extended lymphadenectomy, intra-operative blood loss and sterility of surgery) and oncological variables (neoadjuvant therapy and TNM stage). Therefore, the current evidence does not mean that abdominal drainage should be discontinued in all patients after GC surgery. What we can conclude is the avoidance of routine drainage of a prophylactic nature. Drainage is strongly recommended in some cases, such as abdominal contamination due to perforation and obvious iatrogenic organ injury [40, 47]. In addition, there is evidence demonstrating that PD may be useful in high-risk patients with long operative time or massive intraoperative bleeding [31, 34].

Recently, the first nomogram for predicting the risk of postoperative percutaneous drain placement has been constructed [31]. This prediction model encompassed sex, age, surgical approach, and operative time, which may enable the surgeons to identify high-risk patients, so that PD can be performed selectively. However, this model was derived from a retrospectively study without external validation. Future multicenter RCTs including risk-stratified randomization are urgently needed before final conclusions can be drawn.

The present study has some limitations that should be acknowledged. First, although 7 RCTs were included in our study, the quality of these RCTs was not high and did not also perform stratified analyses in specific populations, which had a certain impact on the reliability of the results of this study. Therefore, more well-designed RCTs with large sample sizes are expected to provide more credible evidence on this issue. Second, several included studies [24, 26, 33] were published over a large time frame, so improvements in gastric surgery and perioperative management during this time could potentially influence the results. Third, there was considerable heterogeneity between studies, including the type of drain used and the period of drain placement, which could also have an impact on the reliability of our results.

## Conclusions

The present meta-analysis suggests that the routine use of PD after GC surgery is not beneficial, and even harmful with increased morbidities, and prolonged time to soft diet and hospital stay. However, based on the abovementioned limitations and low level of evidence of the comparisons, more multicenter RCTs with risk-stratified randomization are needed to confirm these questions.

## Supplementary Information

**Additional file 1:** **Table S1.** Detailed search strategies of each database. **Table S2.** Outcomes of the meta-regression analyses. **Figure S1.** Forest plots of perioperative outcomes including: A. anastomotic leakage; B. Duodenal stump leakage; C. Pancreatic leakage; D. Intra-abdominal abscess; E. Surgical-site infection; F. Pulmonary infection; G. Mortality; H. Need for additional drainage; I. Readmission; J. Reoperation. **Figure S2.** Subgroup analyses of perioperative outcomes based on sample size (≥100 vs. <100). A. total complications; B. anastomotic leakage; C. Duodenal stump leakage; D. Pancreatic leakage; E. Intra-abdominal abscess; F. Surgical-site infection; G. Pulmonary infection; H. Mortality; I. Time to first soft diet; J. Length of hospital stay; K. Need for additional drainage; L. Readmission; M. Reoperation. **Figure S3.** Subgroup analyses of perioperative outcomes based on academic institution (Yes vs. No). A. total complications; B. anastomotic leakage; C. Duodenal stump leakage; D. Pancreatic leakage; E. Intra-abdominal abscess; F. Surgical-site infection; G. Pulmonary infection; H. Mortality; I. Time to first soft diet; J. Length of hospital stay; K. Need for additional drainage; L. Readmission; M. Reoperation. **Figure S4.** Subgroup analyses of perioperative outcomes in GC patients who underwent laparoscopic surgery. A. total complications; B. anastomotic leakage; C. Duodenal stump leakage; D. Pancreatic leakage; E. Intra-abdominal abscess; F. Surgical-site infection; G. Pulmonary infection; H. Time to first soft diet; I. Length of hospital stay; J. Reoperation. **Figure S5.** Subgroup analyses of perioperative outcomes in GC patients who underwent total gastrectomy. A. total complications; B. anastomotic leakage; C. Duodenal stump leakage; D. Pancreatic leakage; E. Intra-abdominal abscess; F. Surgical-site infection; G. Pulmonary infection; H. Mortality; I. Time to first soft diet; J. Length of hospital stay; K. Need for additional drainage; L. Readmission; M. Reoperation. **Figure S6.** Begg’s funnel plot of perioperative outcomes including: A. total complications; B. anastomotic leakage; C. Intra-abdominal abscess; D. Surgical-site infection; E. Pulmonary infection; F. Time to first soft diet; G. Length of hospital stay. All *P* values >0.05.

## Data Availability

All data generated or analyzed during this study are included in this published article.

## Abbreviations

GC: Gastric cancer
PD: Prophylactic drainage
ND: Non-drainage
RCTs: Randomized controlled trials
ERAS: Enhanced recovery after surgery
GRADE: Grading of Recommendations Assessment, Development, and Evaluation
OR: Odds ratio
MD: Mean difference
HR: Hazard ratio
CI: Confidence interval
SD: Standard deviation
RoB: Cochrane Risk-of-Bias
ROBINS-I: Risk of Bias in Non-Randomized Studies-of Interventions
