# Supplementary material for: Prophylactic drainage versus non-drainage following gastric cancer surgery: a meta-analysis of randomized controlled trials and observational studies
Source: World J Surg Oncol. 2023 Jun 3;21:166. doi: 10.1186/s12957-023-03054-1 (PMC10239187; doi:10.1186/s12957-023-03054-1)
Supplement: Supplementary file 1 — Additional file 1: Table S1. Detailed search strategies of each database. Table S2. Outcomes of the meta-regression analyses. Figure S1. Forest plots of perioperative outcomes including: A. anastomotic leakage; B. Duodenal stump leakage; C. Pancreatic leakage; D. Intra-abdominal abscess; E. Surgical-site infection; F. Pulmonary infection; G. Mortality; H. Need for additional drainage; I. Readmission; J. Reoperation. Figure S2. Subgroup analyses of perioperative outcomes based on sample size (≥100 vs. <100). A. total complications; B. anastomotic leakage; C. Duodenal stump leakage; D. Pancreatic leakage; E. Intra-abdominal abscess; F. Surgical-site infection; G. Pulmonary infection; H. Mortality; I. Time to first soft diet; J. Length of hospital stay; K. Need for additional drainage; L. Readmission; M. Reoperation. Figure S3. Subgroup analyses of perioperative outcomes based on academic institution (Yes vs. No). A. total complications; B. anastomotic leakage; C. Duodenal stump leakage; D. Pancreatic leakage; E. Intra-abdominal abscess; F. Surgical-site infection; G. Pulmonary infection; H. Mortality; I. Time to first soft diet; J. Length of hospital stay; K. Need for additional drainage; L. Readmission; M. Reoperation. Figure S4. Subgroup analyses of perioperative outcomes in GC patients who underwent laparoscopic surgery. A. total complications; B. anastomotic leakage; C. Duodenal stump leakage; D. Pancreatic leakage; E. Intra-abdominal abscess; F. Surgical-site infection; G. Pulmonary infection; H. Time to first soft diet; I. Length of hospital stay; J. Reoperation. Figure S5. Subgroup analyses of perioperative outcomes in GC patients who underwent total gastrectomy. A. total complications; B. anastomotic leakage; C. Duodenal stump leakage; D. Pancreatic leakage; E. Intra-abdominal abscess; F. Surgical-site infection; G. Pulmonary infection; H. Mortality; I. Time to first soft diet; J. Length of hospital stay; K. Need for additional drainage; L. Readmission; M. Reo [file 12957_2023_3054_MOESM1_ESM.docx]

Table S1. Detailed search strategies of each database.

| Database | Search strategy |
| --- | --- |
| Pubmed | (“drainage [Title/ Abstract]” OR “drain [Title/ Abstract]”) AND (“gastric cancer [Title/ Abstract]” OR “gastric carcinoma [Title/ Abstract]” OR “stomach cancer [Title/ Abstract]” OR “stomach neoplasm [Title/ Abstract]”) |
| Embase | (“drainage”: ab, ti OR “drain”: ab, ti) AND (“gastric cancer”: ab, ti OR “gastric carcinoma”: ab, ti OR “stomach cancer”: ab, ti OR “stomach neoplasm”: ab, ti) |
| Web of Science | (“drainage [Abstract]” OR “drain [Abstract]”) AND (“gastric cancer [Abstract]” OR “gastric carcinoma [Abstract]” OR “stomach cancer [Abstract]” OR “stomach neoplasm [Abstract]”) |
| Cochrane Central Register of Controlled Trials | (“drainage [Abstract]” OR “drain [Abstract]”) AND (“gastric cancer [Abstract]” OR “gastric carcinoma [Abstract]” OR “stomach cancer [Abstract]” OR “stomach neoplasm [Abstract]”) |
| China National Knowledge Infrastructure | “胃癌（摘要）”OR “引流（摘要）” |

Table S2. Outcomes of the meta-regression analyses.

|  | Time to first soft diet | | |  | Postoperative hospital stay | | |
| --- | --- | --- | --- | --- | --- | --- | --- |
| Covariates | Standardized β coeffcient | P value | 95 %CI |  | Standardized β coeffcient | P value | 95 %CI |
| Study design | -1.75 | 0.440 | -0.711-0.361 |  | -0.89 | 0.157 | -2.172-0.391 |
| Sample size | -0.60 | 0.451 | -2.489-1.289 |  | -0.73 | 0.605 | -3.708-2.246 |
| Academic institution | -4.55 | 0.344 | -1.573-0.663 |  | -0.01 | 0.990 | -1.355-1.340 |
| Surgical approach | -1.77 | 0.087 | -3.897-0.366 |  | -1.82 | 0.164 | -4.499-0.850 |
| Surgical procedure | -7.78 | 0.483 | -3.419-1.863 |  | 0.34 | 0.711 | -1.584-2.258 |


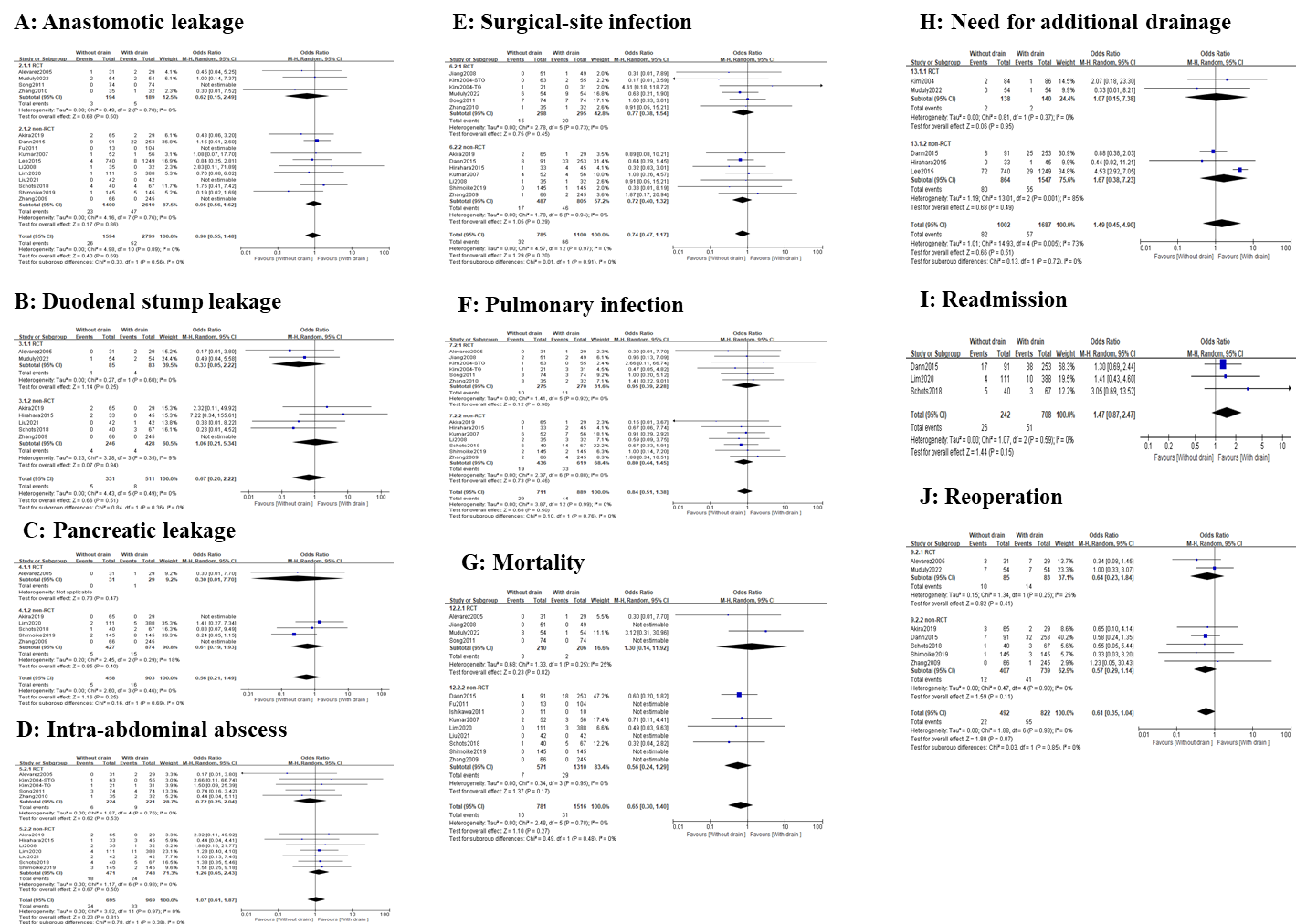


Figure S1. Forest plots of perioperative outcomes including: A. anastomotic leakage; B. Duodenal stump leakage; C. Pancreatic leakage; D. Intra-abdominal abscess; E. Surgical-site infection; F. Pulmonary infection; G. Mortality; H. Need for additional drainage; I. Readmission; J. Reoperation.


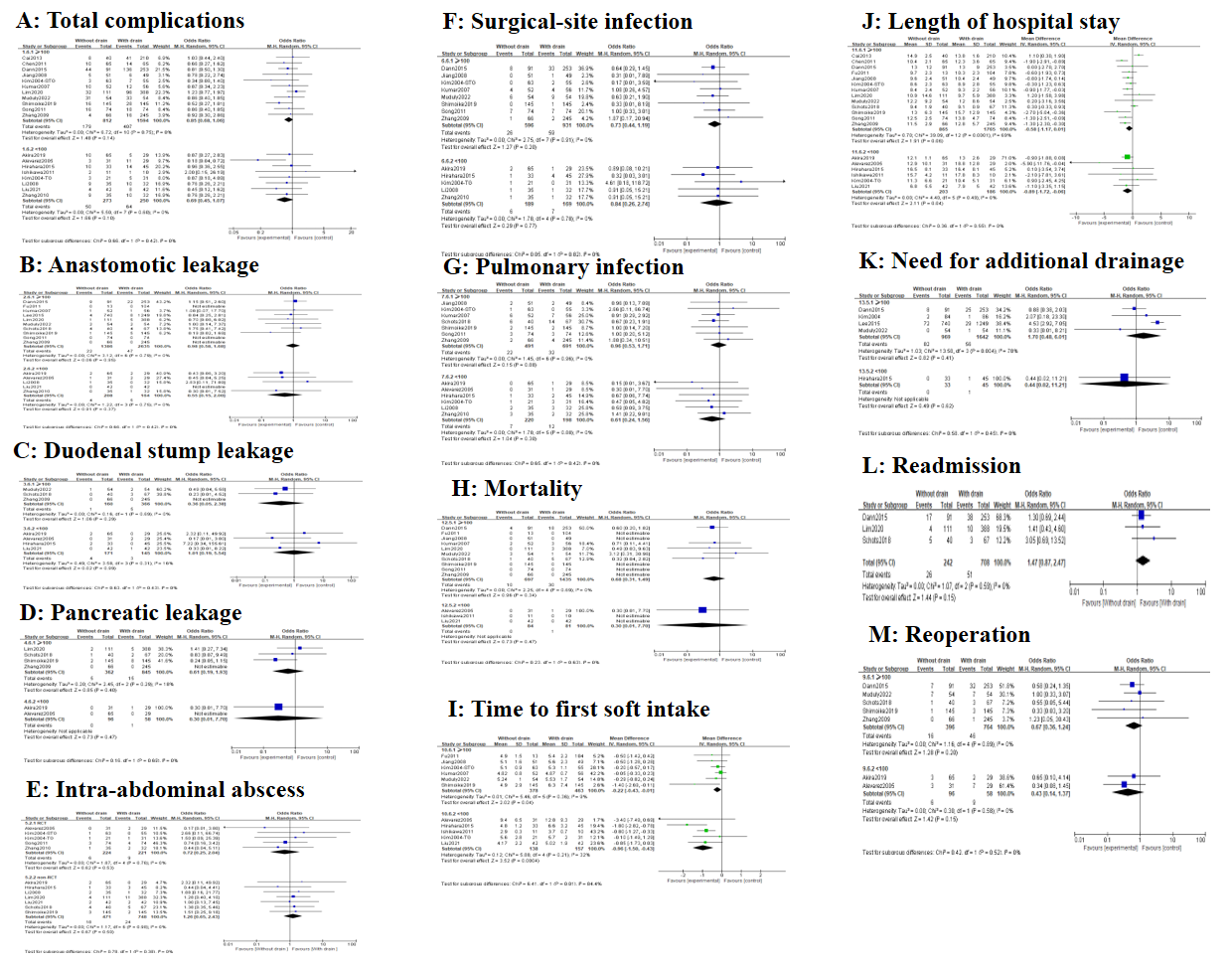


Figure S2. Subgroup analyses of perioperative outcomes based on sample size (≥100 vs. <100). A. total complications; B. anastomotic leakage; C. Duodenal stump leakage; D. Pancreatic leakage; E. Intra-abdominal abscess; F. Surgical-site infection; G. Pulmonary infection; H. Mortality; I. Time to first soft diet; J. Length of hospital stay; K. Need for additional drainage; L. Readmission; M. Reoperation.


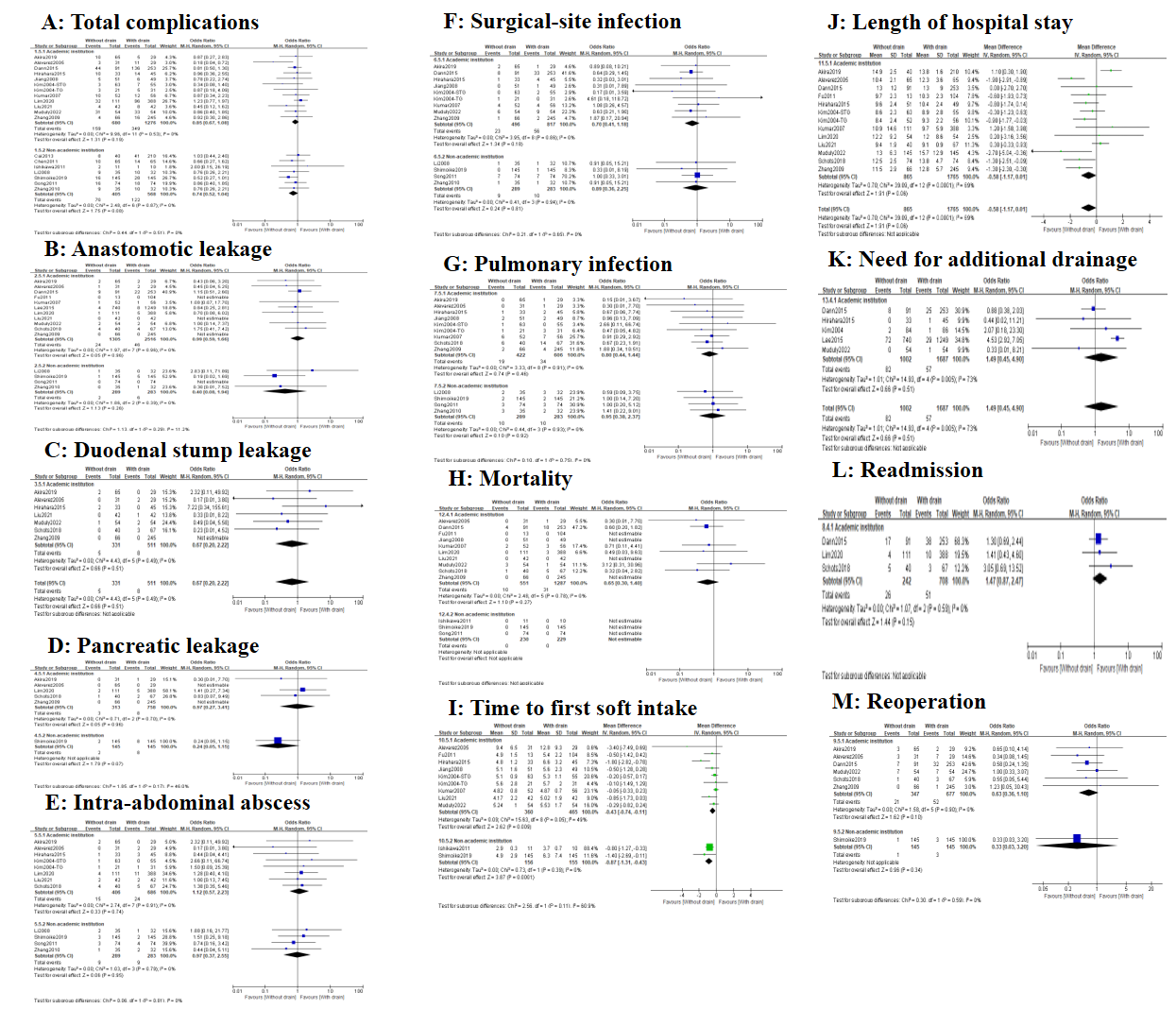


Figure S3. Subgroup analyses of perioperative outcomes based on academic institution (Yes vs. No). A. total complications; B. anastomotic leakage; C. Duodenal stump leakage; D. Pancreatic leakage; E. Intra-abdominal abscess; F. Surgical-site infection; G. Pulmonary infection; H. Mortality; I. Time to first soft diet; J. Length of hospital stay; K. Need for additional drainage; L. Readmission; M. Reoperation.


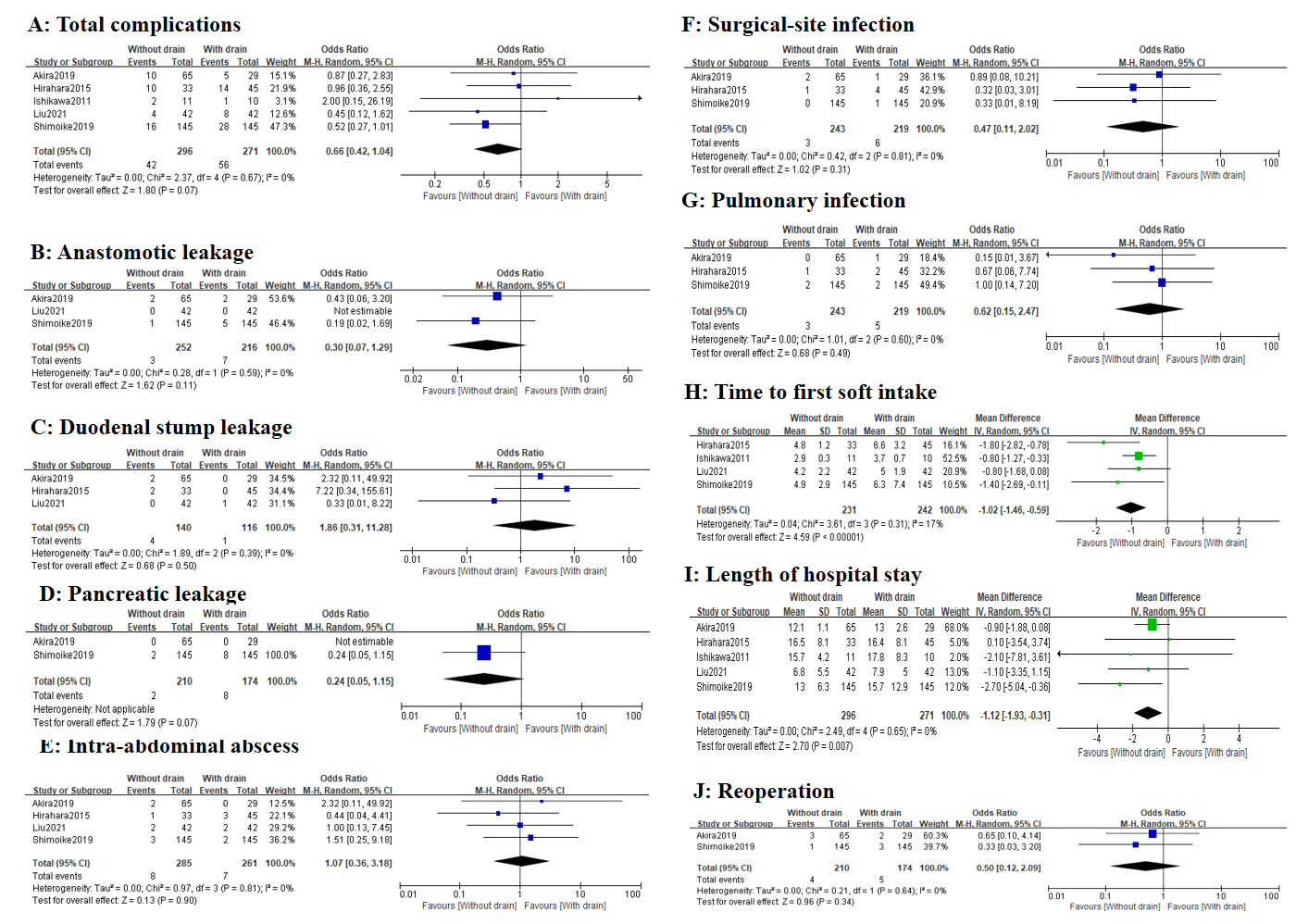


Figure S4. Subgroup analyses of perioperative outcomes in GC patients who underwent laparoscopic surgery. A. total complications; B. anastomotic leakage; C. Duodenal stump leakage; D. Pancreatic leakage; E. Intra-abdominal abscess; F. Surgical-site infection; G. Pulmonary infection; H. Time to first soft diet; I. Length of hospital stay; J. Reoperation.


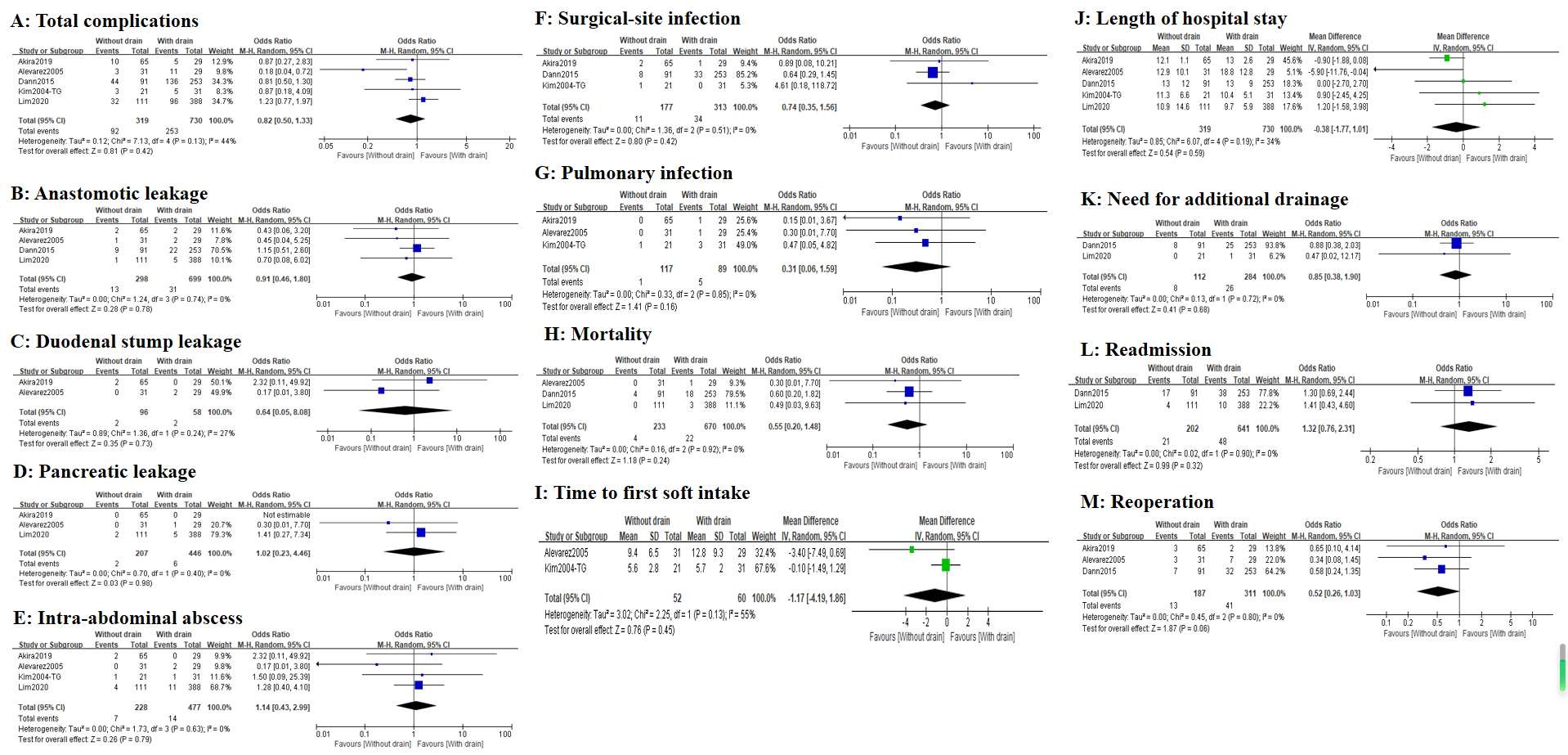


Figure S5. Subgroup analyses of perioperative outcomes in GC patients who underwent total gastrectomy. A. total complications; B. anastomotic leakage; C. Duodenal stump leakage; D. Pancreatic leakage; E. Intra-abdominal abscess; F. Surgical-site infection; G. Pulmonary infection; H. Mortality; I. Time to first soft diet; J. Length of hospital stay; K. Need for additional drainage; L. Readmission; M. Reoperation.


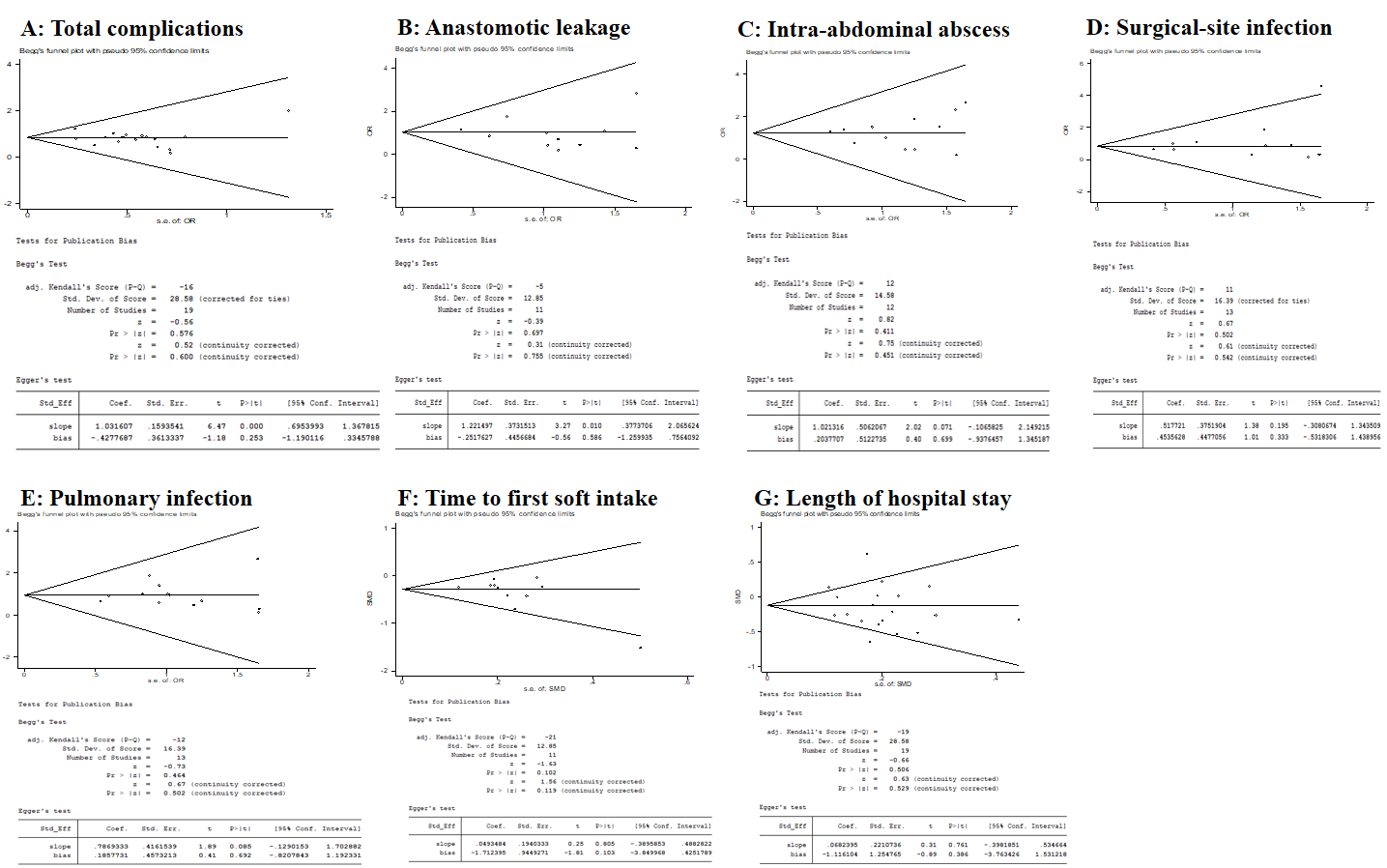


Figure S6. Begg’s funnel plot of perioperative outcomes including: A. total complications; B. anastomotic leakage; C. Intra-abdominal abscess; D. Surgical-site infection; E. Pulmonary infection; F. Time to first soft diet; G. Length of hospital stay. All P values >0.05.
